# Spectrum and Image Texture Features Analysis for Early Blight Disease Detection on Eggplant Leaves

**DOI:** 10.3390/s16050676

**Published:** 2016-05-11

**Authors:** Chuanqi Xie, Yong He

**Affiliations:** College of Biosystems Engineering and Food Science, Zhejiang University, Hangzhou 310058, China; cqxie@zju.edu.cn

**Keywords:** texture feature, hyperspectral imaging, RGB/HSV/HLS image, classification, early blight disease, eggplant

## Abstract

This study investigated both spectrum and texture features for detecting early blight disease on eggplant leaves. Hyperspectral images for healthy and diseased samples were acquired covering the wavelengths from 380 to 1023 nm. Four gray images were identified according to the effective wavelengths (408, 535, 624 and 703 nm). Hyperspectral images were then converted into RGB, HSV and HLS images. Finally, eight texture features (mean, variance, homogeneity, contrast, dissimilarity, entropy, second moment and correlation) based on gray level co-occurrence matrix (GLCM) were extracted from gray images, RGB, HSV and HLS images, respectively. The dependent variables for healthy and diseased samples were set as 0 and 1. K-Nearest Neighbor (KNN) and AdaBoost classification models were established for detecting healthy and infected samples. All models obtained good results with the classification rates (CRs) over 88.46% in the testing sets. The results demonstrated that spectrum and texture features were effective for early blight disease detection on eggplant leaves.

## 1. Introduction

The hyperspectral image is also called hyperspectral cube because it consisted of a series of gray images covering the full wavelengths [1]. It can provide not only spectral reflectance information but also image features at the same time [2]. It is different from multispectral imaging, which can only provide images at several bands. Also, multispectral imaging does not produce spectral information. Based on one hyperspectral image, those gray images with significant information can be obtained. Moreover, RGB images can be acquired from hyperspectral images and then transferred into the images in HSV and HLS color spaces. On the basis of so much useful information, hyperspectral imaging has already been used in many studies, such as food [3,4], agriculture [5], geography [6] and archaeology [7]. However, in most of these studies, only spectral reflectance information was studied. For disease detection, hyperspectral imaging has also been widely applied in many previous studies. Huang *et al.* studied yellow rust in wheat using *in-situ* spectral reflectance and hyperspectral imaging [8]. Mahlein *et al.* used spectral information from hyperspectral images for detecting different diseases (*Cercospora* leaf spot, powdery mildew and leaf rust) on sugar beet leaves [9]. Bulanon *et al.* diagnosed citrus black spot diseased samples from healthy and other diseased samples, such as greasy spot, melanoses and wind scar [10]. However, most of these studies were focused on spectral signature rather than imaging features.

Texture is a significant image feature which corresponds to both brightness value and pixel locations [11]. The texture feature information can reflect the intensity change of pixels, indicating that it contains geometric structure information for the image [12]. Texture feature analysis based on hyperspectral images can also be found in many previous studies [13,14,15,16]. When crops are infected by a disease, some of their features, such as physiological and biochemical indexes and color, may change to some extent, which may directly result in some changes of the texture features. Thus, eight texture features (mean, variance, homogeneity, contrast, dissimilarity, entropy, second moment and correlation) based on gray level co-occurrence martrix (GLCM) were extracted from healthy and infected images and studied in this work. GLCM is a common texture analysis method using the second-order statistics of co-occurrence matrix. In this study, GLCM-based texture features were extracted from gray images, RGB, HSV and HLS images, respectively.

This study investigated both spectrum and texture features for early blight disease detection on eggplant leaves. Early blight is a common fungal disease in eggplant. It can cause decreased quality and yield, especially in an environment with suitable temperature and high humidity. The objectives of this study were: (1) to detect diseased samples using spectral information; (2) to select effective gray images from hyperspectral images for early blight disease detection; (3) to convert RGB images into HSV and HLS color spaces and (4) to compare the performance of gray images, RGB, HSV and HLS images by two different classifiers.

## 2. Materials and Methods

### 2.1. Samples

Hangqie I eggplants were used in this study. This cultivar is usually planted in Hangzhou, Jiaxing and Huzhou (Zhejiang Province, China). The early blight pathogen *Alternaria solani* was cultivated on potato dextrose agar (PDA) media. A round hypha with about 5 mm diameter was removed using a toothpick and put on the surface of each eggplant leaf. All leaves were sprayed with water mist in order to make them covered by the hyphae successfully. Then, inoculated and non-inoculated samples were kept in different growth chambers with 12 h light/dark cycle, respectively. The temperature was set as 24 °C, and the humidity was set as 90.0%. Finally, 130 healthy and 105 early blight leaves were used for collecting hyperspectral images.

### 2.2. Camera and Software

The hyperspectral imaging system, which covers the wavelengths of 380–1023 nm, was used to acquire hyperspectral images. It consisted of an imaging spectrograph (V10E, Specim, Oulu, Finland), a CCD camera (C8484-05, Hamamatsu Photonics, Hamamatsu, Japan), two light sources (Oriel Instruments, Irvine, CA, USA) with 150 W quartz tungsten halogen lamps, a computer, a moving conveyer and a dark box. The spectral resolution for this system is 2.8 nm, and the area CCD array detector has 672 pixels × 512 bands (spatial × spectral). ENVI 5.2 (Research System Inc., Boulder, CO, USA) and MATLAB R2014a (The Math Works Inc., Natick, MA, USA) were used to process and analyze the data.

### 2.3. Hyperspectral Images

After several attempts, the exposure time was set as 0.13 s, the moving speed was 2.2 mm/s, and the vertical distance between the lens and samples was 35.5 cm. A dark image was acquired by covering the camera lens and turning off the light. A white image was obtained by a Teflon board with the spectral reflectance value of 99%. All leaves were numbered and then tiled on the moving conveyor to be scanned by the camera. When a raw hyperspectral image was generated, it can be corrected by the dark and white images according to Equation (1). For one hyperspectral image, it consisted of 512 gray images covering the wavelengths from 380 to 1023 nm:

(1) $$C=\frac{R-D}{W-D}$$

where *C* is the corrected hyperspectral image, *R* is the raw hyperspectral image, *D* is the dark image, and *W* is the white image.

### 2.4. Conversion from RGB into HSV and HLS Images

**Figure 1:**
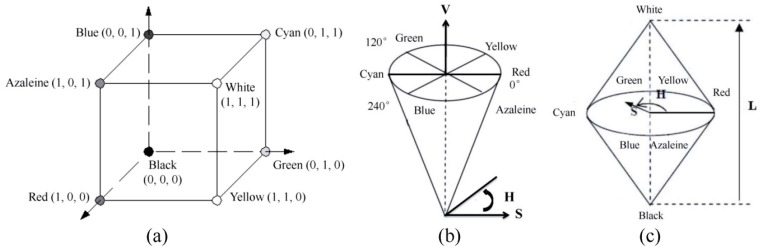
(**a**) RGB color space; (**b**) HSV color space; and (**c**) HLS color space.

The RGB color space is a common color standard in which red (R), green (G), and blue (B) are mixed together in different ways in order to generate a wide array of colors. For each parameter in RGB color space, it ranges from 0 to 255. High value means bright of objective. HSV color space is another color model where H stands for hue, S stands for saturation and V stands for value. The H index ranges from 0° to 360°, 0° means red, 60° indicates yellow, 120° is corresponding to green, 180° means cyan, 240° indicates blue and 300° is corresponding to azaleine. The S covers from 0 to 1, and the higher value means more saturated. V covers from 0 to 255 in which 0 represents for black and 255 represents for white. In HLS color space, H means hue, L means lightness and S means saturation. The two Hs have the same meaning in HSV and HLS color spaces. When H value equals to 0, the image is gray image. L is located from 0 to 1 where 0 indicates black and 1 indicates white. S also covers from 0 to 1 in which a higher value means more bright-colored. The three color spaces were shown in Figure 1.

However, the three different color spaces can be transferred into each other. The conversion method can be described as follows: suppose that *X* is the maximum value among R, G and B, and *Y* is the minimum one. Thus, the HSV color space can be described as:

(2) $$H=\left\{ \begin{array}{ll} 0^{\circ } & \mathrm{if}\;X=Y\\ 60^{\circ }\times \frac{G-B}{X-Y}+0^{\circ }, & \mathrm{if}\;X=R\;\mathrm{and}\;G\geq B\\ 60^{\circ }\times \frac{G-B}{X-Y}+360^{\circ }, & \mathrm{if}\;X=R\;\mathrm{and}\;G<B\\ 60^{\circ }\times \frac{B-R}{X-Y}+120^{\circ }, & \mathrm{if}\;X=G\\ 60^{\circ }\times \frac{R-G}{X-Y}+240^{\circ }, & \mathrm{if}\;X=B \end{array}\right.$$

(3) $$S=\left\{ \begin{array}{ll} 0 & \mathrm{if}\;X=0\\ \frac{X-Y}{X}=1-\frac{Y}{X}, & otherwise\; \end{array}\right.$$

(4) V=X

The HLS color model can be described as:

(5) $$H=\left\{ \begin{array}{ll} 0^{\circ } & \mathrm{if}\;X=Y\;\\ 60^{\circ }\times \frac{G-B}{X-Y}+0^{\circ }, & \mathrm{if}\;X=R\;\mathrm{and}\;G\geq B\\ 60^{\circ }\times \frac{G-B}{X-Y}+360^{\circ }, & \mathrm{if}\;X=R\;\mathrm{and}\;G<B\;\\ 60^{\circ }\times \frac{B-R}{X-Y}+120^{\circ }, & \mathrm{if}\;X=G\\ 60^{\circ }\times \frac{R-G}{X-Y}+240^{\circ }, & \mathrm{if}\;X=B \end{array}\right.$$

(6) $$L=\frac{X+Y}{2}$$

(7) $$S=\left\{ \begin{array}{ll} 0^{\circ } & \mathrm{if}\;L=0\;\mathrm{or}\;X=Y\;\\ \frac{X-Y}{X+Y}=\frac{X-Y}{2L}, & \mathrm{if}\;0<L<0.5\;\\ \frac{X-Y}{2-(X+Y)}=\frac{X-Y}{2-2L}, & \mathrm{if}\;L>0.5\; \end{array}\right.$$

### 2.5. Texture Features Based on GLCM

Texture features can reflect the relationship of neighboring pixels in one image [14]. In this study, eight different texture features (mean, variance, homogeneity, contrast, dissimilarity, entropy, second moment and correlation) based on GLCM were extracted from each image. Different features stand for different relationships among the various pixels. The description of the texture features can be seen in Table 1 [17,18].

### 2.6. Regression Coefficients

In this study, gray images were identified at the four wavelengths suggested by the regression coefficients (RC) method. The size of the coefficients (the absolute value of RC) gives an indication of which wavelengths have a significant impact on the response variables [19]. The high positive and negative peaks represent the wavelengths at these points contain more useful information [20].

### 2.7. Classifiers and Evaluation Performance

#### 2.7.1. Principal Component Analysis

Principal component analysis (PCA) is a powerful method for data compression and effective information selection in hyperspectral imaging analysis [21]. It can extract useful information, decrease the noise and reduce the number of original variables. It summarizes data by forming new variables, which are linear composites of the original variables. The new variables (principal components, PCs) are uncorrelated, which can represent the raw data. Based on these PCs, the main information can be obtained, which is convenient for image analysis [22]. In this study, raw data with *n* × *m* dimension (*n* is the number of samples and *m* is the number of wavebands or texture values) was reduced into the data with *n* × *p* dimension (*n* is the number of samples and *p* is the number of PCs). In this way, the most useful information can be compressed into only several PCs. According to the accumulative contribution rate, the first several PCs were identified.

#### 2.7.2. K-Nearest Neighbor and AdaBoost

K-Nearest Neighbor (KNN) is a kind of a classification method, which uses the majority voting rule, considering a weighted vote of the KNNs of the training set [23]. It is often used for identifying which category the unknown samples should belong to. This method can usually acquire good classification performance. The principle is: suppose the sample *i* is known, all of other samples which are neighboring or similar with this sample should be found out and treated as the training set. Then, the category for sample *i* can be identified according to the feature of the training set. Each sample is calculated and classified into its specific set.

The AdaBoost classifier method combines many linear weak classifiers together, turning out to be very effective in pattern recognition and machine learning fields [24]. In the AdaBoost classifier, each weak classifier only operates the classification for one dimension, which may not be excellent [25]. However, all weak classifiers can be combined into be a perfect one by determining their fusion weights [26,27]. For the AdaBoost algorithm, all samples are originally allocated equal weights. Some samples which are classified correctly by the weak classifier can get lower weights while others that are classified incorrectly obtain higher weights [28]. The same operation is repeated many times and a range of weak classifiers is generated. Thus, those samples which are misclassified previously are re-weighted, resulting in more samples being identified correctly. Based on this principle, the total classification result can be improved. This method does not expect all classifiers to be excellent but it does expect each one should contribute in order to get a correct classification result for the hard sample [26].

#### 2.7.3. Model Evaluation

Performance of the classification models were evaluated based on the values of classification rates (CRs), which should be between 0% and 100%. The CR was calculated by the ratio of correct identification samples and the total samples. The higher the CR value, the better the classification result. In this study, the CR values in both training and testing sets were measured. For each type of samples (healthy and diseased), the classification results were also compared and discussed.

### 2.8. Study Flow

**Figure 2:**
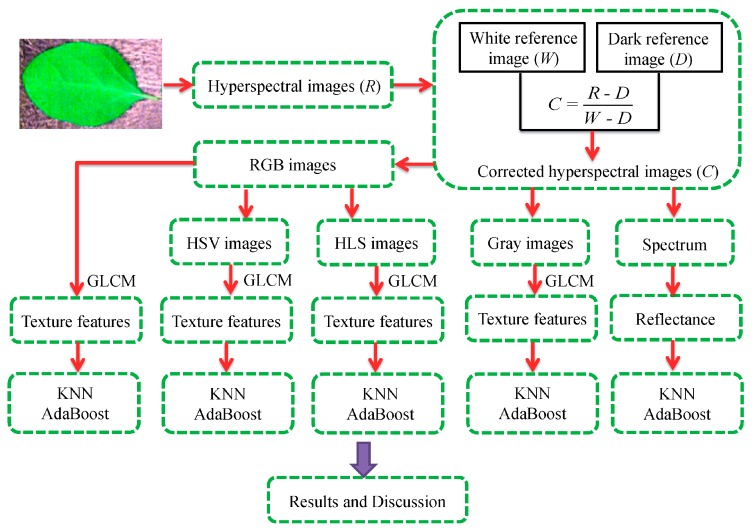
The flow diagram of this study.

The flow diagram of this study was shown in Figure 2. Raw hyperspectral images of eggplant leaves were acquired by the hyperspectral camera firstly, then, they were corrected by the dark and white images. Spectral reflectance was extracted from corrected hyperspectral images. Gray images at the effective wavelengths were identified. Similarity, RGB images were obtained from the corrected hyperspectral images. Also, RGB images were transferred into those images in HSV and HLS color spaces, respectively. For these gray images, RGB, HSV and HLS images, texture features based on GLCM were extracted and treated as the independent variables. Finally, two classification models (KNN and AdaBoost) were established for detecting the diseased samples.

## 3. Results and Discussion

### 3.1. Results Based on Spectrum

#### 3.1.1. Spectral Reflectance

**Figure 3:**
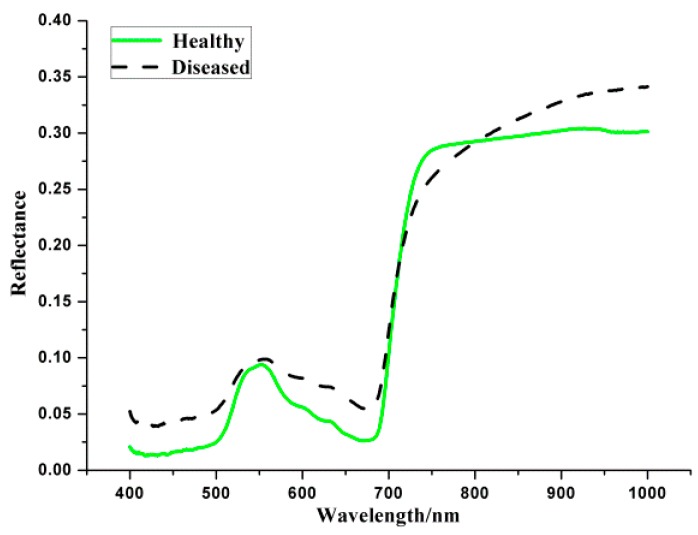
Average spectral reflectance curves of healthy and infected samples.

The average spectral reflectance curves can be seen in Figure 3. It can be found that the general trends of the two curves are very similar. The wavelength at 555 nm is the nitrogen absorption band, and the wavelength at 970 nm is the water absorption band. However, there are obvious differences between healthy and diseased curves. That might be the reason why spectral reflectance can be used for the classification of different samples. Due to the noise at the beginning and the end of the wavelength, only the 400–1000 nm range (477 wavebands) was studied.

#### 3.1.2. Distribution Based on PCA

**Figure 4:**
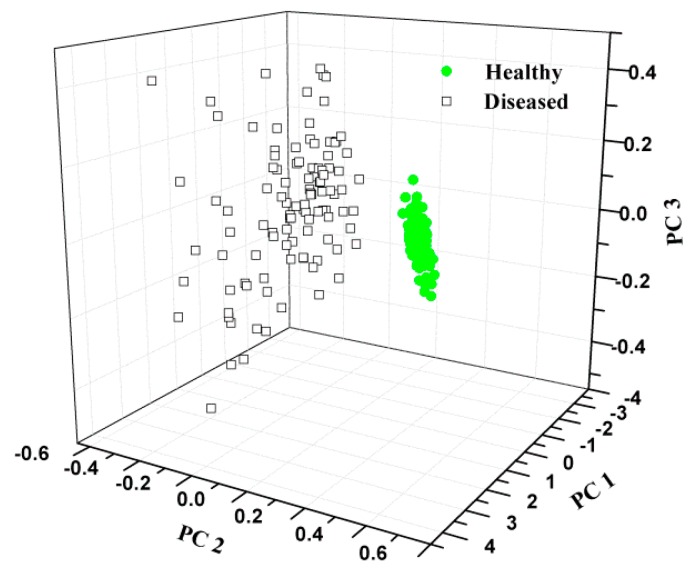
Samples distribution of PCA based on the spectrum.

Since the raw data has 477 input variables, PCA was firstly carried out to compress the original data and extract the most useful information. Based PCA calculation, three PCs were obtained, which explained 91.12%, 6.60% and 1.95% variance, indicating the three PCs could stand for most of the variance. The scores scatter plot of the first three PCs can be seen in Figure 4. There was an obvious boundary between the two types.

#### 3.1.3. Classification Results

The full variables were used to establish KNN and AdaBoost classification models. They obtained excellent results in each classifier (Table 2). All samples were identified correctly in KNN model, and only one sample was misidentified in AdaBoost model. However, too many input variables increased the calculation time and also might affect the robust of the model due to the redundant information.

### 3.2. Gray Images

**Figure 5:**
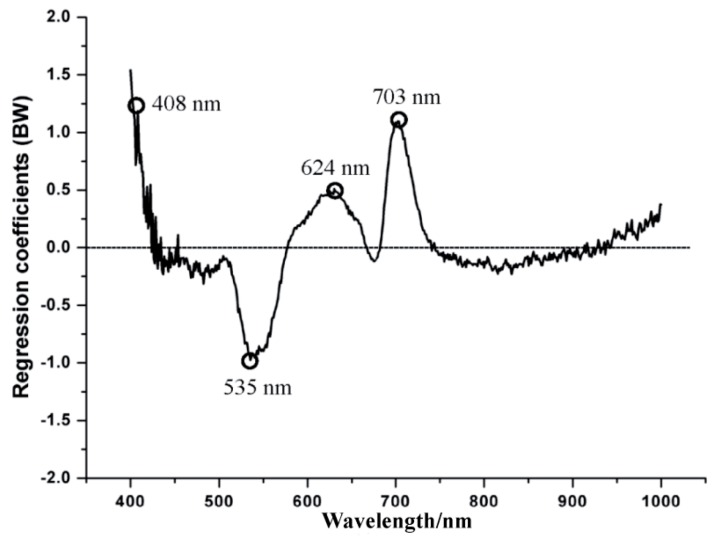
Effective wavelengths selected by regression coefficients.

The RC method was firstly used to select useful wavelengths, resulting in the corresponding gray images. Four wavelengths were obtained (408, 535, 624 and 703 nm), which can be seen in Figure 5.

**Figure 6:**
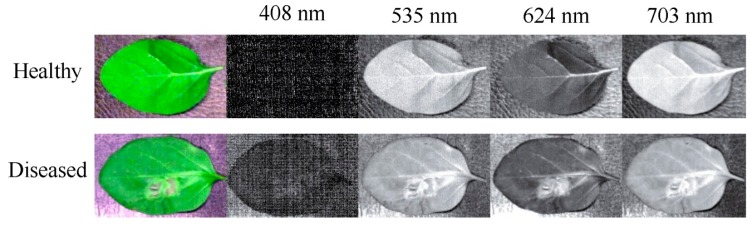
Gray images extracted from hyperspectral images.

In Figure 5, the large absolute values indicated that the wavelengths at these points contain more useful information [29]. Thus, four gray images at these wavelengths were identified. The selected gray images were shown in Figure 6. Compared with 512 gray images for the whole spectral wavelengths, the number of new images only accounted for 0.78%. Then, the texture features were extracted from the four images.

### 3.3. RGB, HSV and HLS Images

**Figure 7:**
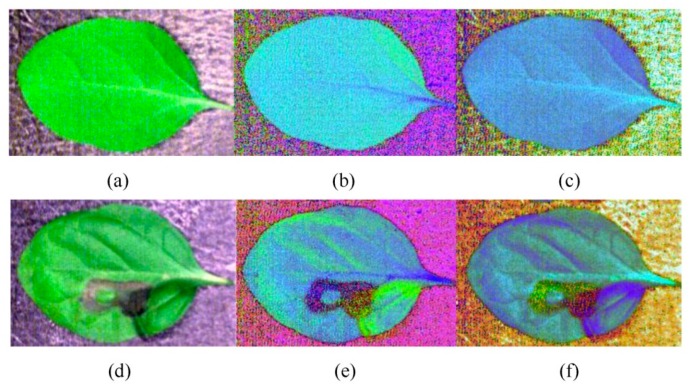
The healthy image in (**a**) RGB; (**b**) HSV and (**c**) HLS color spaces; the diseased image in (**d**) RGB; (**e**) HSV and (**f**) HLS color spaces.

RGB, HSV and HLS images for healthy and diseased samples were shown in Figure 7a–f. It can be seen that healthy and diseased areas are different in RGB, HSV and HLS color spaces, respectively. Even for the same sample, it showed differently in the three color spaces.

### 3.4. Results Based on Gray Images

#### 3.4.1. Texture Images

**Figure 8:**
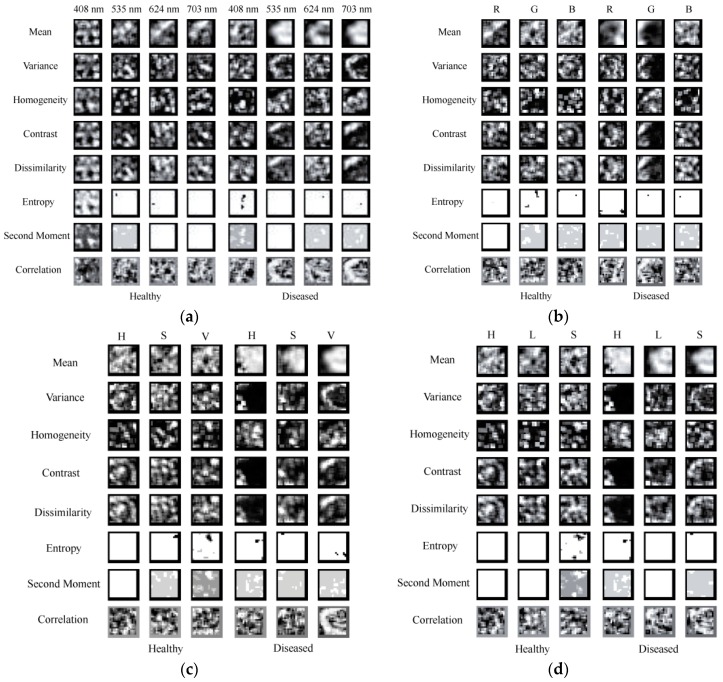
Texture images extracted from (**a**) gray images; (**b**) RGB images; (**c**) HSV images and (**d**) HLS images.

Figure 8a showed different texture images for both healthy and diseased samples at the wavelengths of 408, 535, 624 and 703 nm. It can be seen that for the same area and wavelength, texture images are different due to the different texture features; for the same area and texture feature, texture images vary for different wavelengths; for the same texture and wavelength, texture images are also different because of the various areas. This may be the reason why texture features can be used for classifying healthy and diseased samples.

#### 3.4.2. Distribution Based on PCA

**Figure 9:**
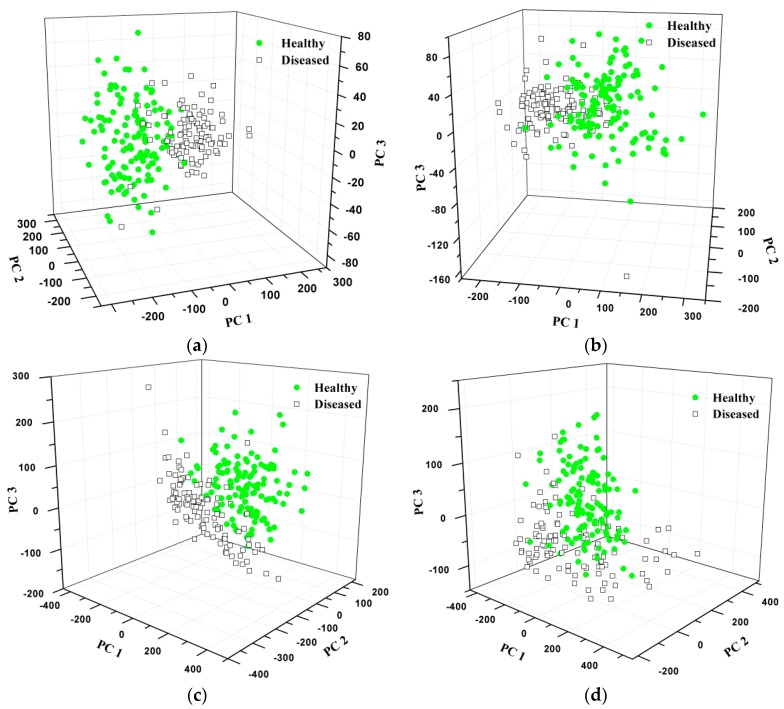
Samples distribution of PCA based on (**a**) gray images; (**b**) RGB images; (**c**) HSV images; and (**d**) HLS images.

PCA was carried out to get the PCs from the texture features. The raw data with 32 independent variables was reduced into three PCs for each sample. For each RGB, HSV or HLS image, the original data has 24 independent variables, and it was changed into three (PCs) after PCA. The scores scatter plot of the first three PCs can be found in Figure 9a. In this study, PC1, PC2 and PC3 accounted for 64.91%, 26.50% and 3.99% variance, respectively, which meant the first three PCs could explain most of the variance (95.39%). It can be seen that most of the healthy and diseased samples distributed in different areas. However, there were still some overlaps between the two different types of samples.

#### 3.4.3. Classification Results

KNN and AdaBoost classification models were established based on the texture features, respectively. The eight texture features at the four wavelengths (8 texture features × 4 wavelengths) were treated as the input variables for the classification models. As can be seen in Table 3, each model performed excellently. The total CRs for KNN model were 100% in the training set and 94.87% in the testing set, respectively. The total results for AdaBoost model were 100% in the training set and 98.72% in the testing set, respectively. For the two models, AdaBoost performed better than KNN. In both models, the classification results for healthy samples were better than those for diseased samples.

### 3.5. Results Based on RGB Images

#### 3.5.1. Texture Images

Figure 8b displayed the texture images obtained from RGB images for healthy and diseased samples. RGB images have three channels, which are R, G and B. In this study, the eight texture features were extracted at each channel, respectively. Thus, a total of 24 values (8 texture features × 3 channels) can be acquired for each image. It can be seen that different texture features, channels and areas are corresponding to different texture images.

#### 3.5.2. Distribution Based on PCA

For the scores of the texture features acquired from RGB images, PC1, PC2 and PC3 represented for 66.76%, 24.80% and 7.09% variance, and the first three PCs could explain 98.65% of the total variance. The samples distribution based on the first three PCs can be seen in Figure 9b. The two types of samples were located in different areas roughly. However, some healthy and diseased samples were mixed together.

#### 3.5.3. Classification Results

KNN and AdaBoost models were then built based on the texture features suggested by RGB images. The results were shown in Table 4. The CRs for the training sets in both models were 100%. In the testing set of KNN model, the CRs for healthy, diseased and all samples were 93.02%, 91.43% and 92.31%, respectively. While in the testing set of AdaBoost model, the three values were 97.67%, 100% and 98.72%, respectively. For the two classifiers, it was also the AdaBoost performed better. In KNN model, the classification result for healthy samples was better than that for diseased samples. The diseased samples performed better than healthy ones in AdaBoost model. Compared with KNN model established based on gray images, the CRs in RGB-KNN model were a little lower. The results in gray images-based AdaBoost model and RGB-AdaBoost model were very similar.

### 3.6. Results Based on HSV Images

#### 3.6.1. Texture Images

The images in the HSV color space were then achieved from RGB images. The HSV also has three channels (H, S and V), and each channel stands for a different meaning. The eight texture features were then extracted from HSV images, resulting in 24 values (8 texture features × 3 channels) shown in Figure 8c. For different texture features, channels and areas, they have various texture images. For the same texture feature and area, the texture images were also different from those of RGB images.

#### 3.6.2. Distribution Based on PCA

The texture features extracted from HSV images were then calculated by PCA. The first three PCs could explain 98.46% of the variance (PC1: 67.55%, PC2: 17.07% and PC3: 13.84%). The three-dimensional space for samples distribution was shown in Figure 9c. This figure showed that some diseased samples can be classified properly while some cannot.

#### 3.6.3. Classification Results

The CRs of HSV images-based KNN and AdaBoost classifiers were shown in Table 5. The overall results for the KNN classifier were 100% in the training set and 93.59% in the testing set, respectively. The result for the AdaBoost model was 100% in each set. It can be found that AdaBoost model could get higher CRs than KNN model. In KNN classifier, the CR for healthy samples was worse than that for diseased samples. While the classification results for healthy and diseased samples were the same in AdaBoost model. Compared with KNN model established based on gray images, the results in HSV-KNN model were a little lower, in which only one more sample was classified incorrectly. However, it performed better than the KNN model built by RGB images. No matter compared with the AdaBoost models based on gray image or RGB images, the results in HSV-AdaBoost model were the best (100%).

### 3.7. Results Based on HLS Images

#### 3.7.1. Texture Images

HLS is another type of color model, which is different from both RGB and HSV color spaces. However, the H and S parameters have the same meanings for HLS and HSV color spaces. This study also attempted to evaluate the performance of texture features extracted from HLS images. Each texture image for different channels, texture features and areas can be seen in Figure 8d. For each HLS image, 24 texture images can be gotten. Similarity, the texture images were decided by the type of texture feature, channel, area and color space. It can be found the texture images in HLS color space were different compared with those in RGB and HSV color spaces.

#### 3.7.2. Distribution Based on PCA

In order to acquire the samples distribution based on HLS images, the texture features were also processed using PCA. The first three PCs could also interpret most of the variance (98.76%). For each PC, the first three variances were 72.17%, 14.79% and 11.80%, respectively. Therefore, the samples distribution in the PCA space could be achieved, which was shown in Figure 9d. However, the performance was not very good.

#### 3.7.3. Classification Results

For the purpose of evaluating the performance of different color spaces and demonstrating texture features extracted from these images can be used to detect early blight disease on eggplant leaves, HLS images were finally used to establish classification models. In Table 6, it is obviously that AdaBoost acquired better results than KNN. For both classifiers, healthy samples performed more excellently than diseased ones. As those models mentioned above, the CRs in the training sets of HLS images-based models were also 100%. However, the total result in the testing set of HLS-KNN model was only 88.46%, which was the lowest one in all models. For HLS-AdaBoost classifier, the classification result was also the lowest (97.44%) compared with other AdaBoost classifiers. However, the results were acceptable. From above analysis, it can be seen that only HLS-KNN model gave a relatively low classification result of 88.46%, while all the other models got CRs over 90%. The results indicated that texture features extracted from gray images, RGB, HSV and HLS images can be used for classifying healthy and early blight diseased eggplant leaves. Also, the two classification models (KNN and AdaBoost) performed better than PCA classification.

### 3.8. Comparison of Different Images

The results in the testing sets of the two classifiers based on different types of images can be seen in Table 7. For the KNN model, the gray image performed the best with the highest classification result (94.87%), while in AdaBoost it was HSV image that obtained the best classification result (100%). In both classifiers, the HLS image got the lowest results (88.46% and 97.44%, respectively). For each type of image, AdaBoost performed better that KNN model. In general, more healthy samples can be identified correctly. All the results demonstrated that image features extracted from hyperspectral images can be used for early blight disease detection.

## 4. Conclusions

In this study, all models provided good results with CRs over 88.46%. The results demonstrated that: (1) the spectral reflectance was useful for classifying healthy and diseased samples; (2) the gray images at the wavelengths of 408, 535, 624 and 703 nm can be used for detecting early blight diseased samples; (3) RGB, HSV and HLS images were also effective for classifying different samples; (4) texture features based on GLCM extracted from gray images, RGB, HSV and HLS images can be treated as input variables for establishing excellent classification models; (5) the two classifiers (KNN and AdaBoost) performed better than PCA; (6) the AdaBoost model gave better performance than the KNN model. From the classification results, it can be found that HLS images performed a little worse, while other types of images can get good results. Thus, those spectrum and texture features extracted from hyperspectral images can be applied in early blight disease detection. In future studies, the performance of each texture feature and other types of texture features should be considered.

## Figures and Tables

**Table 1:** Description of different texture features by gray level co-occurrence matrix (GLCM).

Textures	Description
Mean	The Mean is the average grayscale of all pixels for the image.
Variance	The Variance stands for the change of grayscale.
Homogeneity	The Homogeneity can measure the uniformity of local grayscale for one image. More uniform of the local grayscale means high value of Homogeneity.
Contrast	The Contrast stands for the clarity of texture. The higher the contrast value, the clearer the image.
Dissimilarity	The Dissimilarity mainly represents for the difference of grayscale.
Entropy	The Entropy is the measurement of the information for one image. No texture feature means Entropy value is 0.
Second Moment	The Second Moment reflects the uniformity degree of grayscale.
Correlation	The Correlation can measure the level of similarity in the row or column direction.

**Table 2:** Classification results based on spectral information.

Models	Variables	Training			Testing		
		No. ^1^	Correct	CR/% ^2^	No. ^1^	Correct	CR/% ^2^
KNN	477	157	157	100	78	78	100
AdaBoost	477	157	157	100	78	77	98.72

^1^ Number of samples; ^2^ Classification rate.

**Table 3:** Classification results based on gray images.

Models	Type	Variables	Training			Testing		
			No. ^1^	Correct	CR/% ^2^	No. ^1^	Correct	CR/% ^2^
KNN	Healthy	32	87	87	100	43	42	97.67
	Diseased		70	70	100	35	32	91.43
	All		157	157	100	78	74	94.87
AdaBoost	Healthy	32	87	87	100	43	43	100
	Diseased		70	70	100	35	34	97.14
	All		157	157	100	78	77	98.72

^1^ Number of samples; ^2^ Classification rate.

**Table 4:** Classification results based on RGB images.

Models	Type	Variables	Training			Testing		
			No. ^1^	Correct	CR/% ^2^	No. ^1^	Correct	CR/% ^2^
KNN	Healthy	24	87	87	100	43	40	93.02
	Diseased		70	70	100	35	32	91.43
	All		157	157	100	78	72	92.31
AdaBoost	Healthy	24	87	87	100	43	42	97.67
	Diseased		70	70	100	35	35	100
	All		157	157	100	78	77	98.72

^1^ Number of samples; ^2^ Classification rate.

**Table 5:** Classification results based on HSV images.

10	Type	Variables	Training			Testing		
			No. ^1^	Correct	CR/% ^2^	No. ^1^	Correct	CR/% ^2^
KNN	Healthy	24	87	87	100	43	39	90.70
	Diseased		70	70	100	35	34	97.14
	All		157	157	100	78	73	93.59
AdaBoost	Healthy	24	87	87	100	43	43	100
	Diseased		70	70	100	35	35	100
	All		157	157	100	78	78	100

^1^ Number of samples; ^2^ Classification rate.

**Table 6:** Classification results based on HLS images.

Models	Type	Variables	Training			Testing		
			No. ^1^	Correct	CR/% ^2^	No. ^1^	Correct	CR/% ^2^
KNN	Healthy	24	87	87	100	43	40	93.02
	Diseased		70	70	100	35	29	82.86
	All		157	157	100	78	69	88.46
AdaBoost	Healthy	24	87	87	100	43	43	100
	Diseased		70	70	100	35	33	94.29
	All		157	157	100	78	76	97.44

^1^ Number of samples; ^2^ Classification rate.

**Table 7:** Comparison of the testing results based on different types of images.

Clsssifier	Type	Healthy/% (KNN/AdaBoost)	Diseased/% (KNN/AdaBoost)	All/% (KNN/AdaBoost)	Type	Clsssifier
KNN	Gray image	97.67/100	91.43/97.14	94.87/98.72	Gray image	AdaBoost
	RGB image	93.02/97.67	91.43/100	92.31/98.72	RGB image	
	HSV image	90.70/100	97.14/100	93.59/100	HSV image	
	HLS image	93.02/100	82.86/94.29	88.46/97.44	HLS image	
